# Probing Voltage Dependence Interaction of Cationic Peptides with Bacterial Porins at A Single-Molecule Level

**DOI:** 10.1007/s12013-026-02148-3

**Published:** 2026-08-14

**Authors:** Sonal Prasad

**Affiliations:** 1 Department of Life Sciences and Chemistry, Jacobs University Bremen, Campus Ring 1, 28759 Bremen, Germany; 2 Department of Biomedicine and Clinical Sciences, Linköping University, Linköping, SE-581 83 Sweden

**Keywords:** OmpF porin, Protamine, cationic peptides, single-molecule

## Abstract

This study examined how cationic α‑helical antimicrobial peptides such as protamine exchange between the aqueous phase and the transmembrane β‑barrel porin OmpF (*E. coli*) at the single‑molecule level. High‑resolution conductance recordings in planar lipid bilayers revealed clear voltage‑ and concentration‑dependent ion current blockages, consistent with transient peptide interactions within the OmpF lumen. Peptide length influenced the characteristics of these interactions: longer protamine fragments produced weaker and less frequent blockages. At low transmembrane potentials, all peptides interacted weakly with the pore, whereas higher potentials increased the likelihood and duration of detectable events. These findings establish OmpF as a tractable system for probing cationic peptide–channel interactions and provide a descriptive framework for understanding how arginine‑rich peptides engage with β‑barrel pores due to likely electrostatic trapping, without implying specific transport pathways or translocation mechanisms.

**Supplementary Information:**

The online version contains supplementary material available at https://doi.org/10.1007/s12013-026-02148-3.

## Introduction

The rise of antimicrobial resistance has intensified the search for novel therapeutic strategies, bringing antimicrobial peptides (AMPs) to the forefront of promising alternatives [1–3]. Among them, cationic antimicrobial peptides (CAPs) exhibit broad-spectrum activity and a relatively low propensity for resistance development. Despite their sequence diversity, CAPs typically share key physiochemical features: short length (12–50 amino acids), a net positive charge (+ 4 to + 6), high hydrophobic content (50–70%), and the ability to form amphipathic α-helices in lipid environments [4, 5].

Protamine (Ptm) is a particularly striking CAP, composed of 21 arginine residues and representing the most highly cationic natural peptide known. With a molecular weight of ~ 4.2 kDa and an isoelectric point of ~ 11–13, Ptm lacks defined secondary structure due to the uniformly distributed positive charges [6].

CAPs are generally thought to kill bacteria by disrupting membranes, causing ion leakage, depolarization, and cell death. Several models describe these processes, including the barrel‑stave, toroidal pore, and carpet mechanism [5, 7]. Selectivity toward prokaryotic cells is largely governed by non‑receptor‑mediated recognition of membrane physicochemical properties [8–10]. However, antimicrobial activity at sub‑lytic concentrations suggests additional intracellular targets, such as inhibition of cell wall synthesis, transcription, translation, nucleic acid synthesis, enzymatic activity, or induction of cytoplasmic aggregation [8, 11–15].

Recent evidence indicates that Ptm can enter certain Gram‑negative bacteria without causing membrane disruption. Consistent with this, Ptm does not perturb reconstituted bacterial membranes composed of native phospholipid mixtures, suggesting a non‑lytic, protein‑mediated uptake mechanism [16]. Porin channels abundant, non‑specific outer‑membrane proteins represent a plausible pathway for such translocation [17].

In *Escherichia coli*, outer membrane protein F (OmpF) is one of the best‑characterized porins, present at > 10⁵ copies per cell. It forms a trimer of 16‑stranded β‑barrels, with loop L3 creating a constriction zone defined by a transversal electric field generated by negatively charged residues (D113, E117) on one side and positively charged residues (R42, R82, R132) on the other [18–20]. In *Salmonella typhimurium*, outer membrane protein D (OmpD) is the most abundant porin, accounting for ~ 1% of total cellular protein. Together with OmpC and OmpF, it contributes to ~ 1 × 10⁵–2 × 10⁵ porins per cell and shows high sequence homology to several E. coli porins, reflecting close evolutionary and functional relationships [21].

The goal of these experiments was to determine how peptide length and charge shape the free‑energy barrier for occupying the OmpF and OmpD pores [22], by resolving peptide-porin interactions at single‑channel resolution. Planar bilayer electrophysiology was used to characterize long, highly cationic peptides such as protamine, allowing analysis of their extended pore‑blocking behavior in OmpF. Automated chip‑based patch‑clamp recordings were applied to shorter α‑helical peptides, providing the high‑resolution temporal sensitivity needed to detect rapid binding events and assess whether electrostatic complementarity could facilitate trapping within the charged porin vestibules.

## Materials and Methods

### Peptides

The long highly cationic peptides used in this study were protamine sulfate (ARRRRSSSRPIRRRRPRRRTTRRRRAGRRRR, Y1 from Clupeine P4505, Mw: 4110.51 Da), poly-lysine (Poly-L-lysine hydrobromide 25988-63-0, Mw: 500–2000 Da) and poly-arginine (Poly-L-arginine hydrochloride 26982-20-7, Mw: 5000–15000 Da), protamine C-terminal end (RAGRRRR). These synthetic peptides were ordered from Sigma-Aldrich AG, Germany. The short cationic peptides used in this study were penta-lysine (H-Lys-Lys-Lys-Lys-Lys-OH acetate salt, 19431-21-1, Mw: 658.88 Da), tetra-arginine (H–Arg–Arg–Arg–Arg–OH acetate salt 26791-46-8, Mw: 642.77 Da) and tri-arginine (H–Arg–Arg–Arg–OH acetate salt 19431-21-1, Mw: 486.54 Da). These synthetic peptides were ordered from Bachem AG, Switzerland.

### Planar Bilayer Lipid Membrane (BLM) Preparation and Single Channel Electrophysiological Recordings

Solvent-free lipid bilayers were prepared using a modified version of the Montal–Mueller technique [23]. A small aperture (~ 60–80 μm in diameter) was created in a 25 μm thick Teflon film using a discharge spark (10–50 kV at 500 kHz) generated by a BD-10 Vacuum Tester. The Teflon film was then sealed with silicone grease between two glass half-cells (2.5 ml each), electrically isolating the aqueous compartments.

Prior to bilayer formation, the aperture was pre-treated with 1 µl of 1% n-hexadecane in n-hexane and allowed to dry for 10 min. Both chambers were subsequently filled with buffer solution (1 M KCl, 20 mM MES, pH 6.0). A lipid bilayer was formed by spreading 1 µl of 1,2-diphytanoyl-sn-glycero-3-phosphocholine (Avanti Polar Lipids, Alabaster, AL) from a 5 mg/mL solution in n-pentane onto the buffer surface. Bilayer formation occurred within 10 min as the solvent evaporated.

Ag/AgCl electrodes were used to record ionic currents, with the cis-side electrode grounded and the trans-side electrode connected to the headstage of an Axopatch 200B amplifier (Axon Instruments, Foster City, CA). Purified, detergent-solubilized porins (1 µl) were added to the cis chamber and inserted into the bilayer by applying a voltage of 150–200 mV. Successful insertion of single porins was confirmed by discrete increases in current.

For porins, the purified wild type OmpF protein from *E. coli* or wild type OmpD from *S. typhimurium* was diluted from a 1.5 mg/ml stock solution in 1% Octyl-POE detergent (Alexis, Lauchringen, Switzerland). Single-channel insertion typically occurred within 15 min, depending on protein concentration [23]. The porin was added to the trans-side intracellular side of the bilayer, while the peptide was introduced on the cis-side extracellular side which led to stronger interaction with higher events/s (Fig. S1A). Adding porin and peptide to the same side was not favorable, as it resulted in minimal interaction and very few detectable events. In contrast, introducing the porin (trans) and peptide (cis) asymmetric addition produced a favorable orientation, evidenced by a significantly 10x higher number of interaction events compared with porin (cis) and peptide (cis) addition to the symmetric side (Fig. S1B-C). Interestingly, when peptide was added to the trans side of the OmpF, positively applied potentials were needed to generate events. Visual inspection of such traces revealed that the peptide was able to reversibly block the channel from both openings. The output signal was filtered using a low-pass Bessel filter at 10 kHz and sampled at 50 kHz [24, 25].

Recordings involving smaller peptides, such as tri-arginine, were performed using automated planar patch-clamp systems with microstructured glass chips. These integrated devices required < 10 µl of solution and enabled rapid fluid exchange [26]. The resulting bilayers exhibited low capacitance and improved signal-to-noise ratios, allowing high-resolution single-channel recordings (See, Port -a- Patch). All experiments were conducted at room temperature. Data analysis was performed using Clampfit software (Axon Instruments, Inc.).

### Determination of Binding Affinities and Kinetics Constants

Artificial lipid bilayers were formed using the BLM technique, into which single OmpF and OmpD channels were reconstituted. The interaction of peptides with the channel was studied using single-channel electrophysiology. This approach enabled the determination of peptide binding kinetics, including association and dissociation rate constants.

Single-channel recordings of the OmpF pore revealed that peptide partitioning kinetics were influenced by two main factors: Increased event frequency with higher transmembrane potentials, indicating enhanced peptide interaction. Reduced event frequency for longer peptides, suggested hindered interaction with the nanometer-scale pore.

In these measurements, key parameters included: Residence time (*τ*_*off*_ *= τ*_*r*_): Duration of peptide-induced blockages. Open channel time (*τ*_*on*_ *= τ*_*o*_): Time between blockage events. Blockage frequency (*ν*_*m*_): Number of blockage events per second per monomer [27].



The partition coefficient Π(P) between the aqueous phase and the pore lumen was derived from the equilibrium association constant (*K*_*f*_), assuming low pore occupancy [28]: Π(P) = *K*_*f*_ [pept]^∗^, where [pept]^∗^ is the effective molar concentration of a single peptide inside the OmpF pore and was calculated based on *N*_*AV*_ and *V*_*barrel*_: [pept]^∗^ = 1/*N*_*AV*_⋅*V*_*barrel*_ = 0.215 M. *N*_*AV*_ is Avogadro’s number and *V*_*barrel*_ is the internal volume of the β-barrel (~ 0.7 × 1.1 nm²).

Peptide interaction with the pore was treated as a simple adsorption–desorption process between the substrate (S) and the pore (P) to obtain chemical kinetic rates, equilibrium binding constants, and related parameters. In cases of strong binding relative to diffusion, the interaction was treated as a reversible reaction: S + P ⇌ SP.

The kinetic model was limited to a descriptive framework and should be viewed only as a preliminary approximation ensuring no mechanistic conclusions were drawn from the fitted parameters and not considered as a definitive mechanistic explanation. The model assumes low pore occupancy and a single dominant binding mode, consistent with the isolated, non‑overlapping blockage events and single‑exponential dwell‑time distributions observed under the experimental conditions. Although porins can support multi‑state binding, no additional resolvable sub‑conductance levels or kinetic components were detected, justifying the use of this minimal scheme. The assumptions hold under the applied voltages and peptide concentrations. The trimeric architecture of OmpF/OmpD, the possibility of multiple occupancy states, voltage‑coupled electrostatic effects, unresolved fast interactions, and potential sub‑conductance states all introduce mechanistic layers that the current simplified model does not capture. This approach provides a practical kinetic description but does not account for potential electro‑diffusion effects, unresolved fast states, or additional binding substates that may occur outside the temporal resolution of the assay.

### Chip-Based Automated Patch Clamp (Port-a-Patch)

To achieve low-noise recordings and enable detection of fast binding events, a planar lipid bilayer with minimal capacitance was formed. Giant unilamellar vesicles (GUVs) were prepared using the electroformation method [26, 29] in an indium tin oxide (ITO)–coated glass chamber connected to the Vesicle Prep Pro system (Nanion Technologies). The ITO layers on the glass slides served as electrodes due to their electrical conductivity.

A lipid solution containing 5 mM DPhPC and 10% (mol/mol) cholesterol in chloroform was deposited onto the ITO-coated glass surface. Electroformation parameters (amplitude, frequency, duration, etc.) were controlled via the Vesicle Control software (Nanion Technologies).

Purified wild-type OmpF (1.5 mg/ml in 1% octyl-POE) was reconstituted into GUVs as previously described [30]. The final porin concentration was adjusted to 10–20 nM, with detergent reduced to 0.001%. To remove octyl-POE, Bio-Beads were added, and the mixture was incubated overnight at 4 °C. Bio-Beads were subsequently removed by centrifugation. The resulting proteo-GUVs were stored at 4 °C which remained stable for up to one week.

For bilayer formation, 2–3 µl of the proteoliposome suspension was applied to a microstructured borosilicate glass chip (Port-a-Patch, Nanion Technologies) containing a ~ 1 μm aperture. Electrophysiological recordings were performed using the Port-a-Patch automated patch-clamp system. Measurements were conducted in 1 M KCl, 20 mM MES, pH 6.0.

Single-channel currents were recorded using an Axopatch 200B amplifier (Axon Instruments) connected to Ag/AgCl electrodes. Signals were filtered with a 4-pole low-pass Bessel filter at 10 kHz and digitized at 50 kHz using a Digidata 1440 A digitizer. Peptides were added to the cis side of the bilayer, and ion current blockages were monitored to detect binding events [31].

## Results

High-resolution ion conductance current fluctuation measurements were used to quantify peptide-channel interactions using a single trimeric porin reconstituted into a planar lipid bilayer in the presence of the peptide added to either the cis or trans side of the channels.

A total of 70 independent bilayers were formed and 2 pores OmpF and OmpD were analyzed. Reproducibility for each peptide was: Ptm-OmpD- 2, Ptm-OmpF- 10, Poly-arginine- 1, Poly-lysine- 4, Ptm C-terminal- 2, Penta-lysine- 10, Tetra-arginine- 3, Tri-arginine- 2 (Chip-based).

### Interaction of Protamine with OmpF and OmpD Channels

**Fig. 1:**
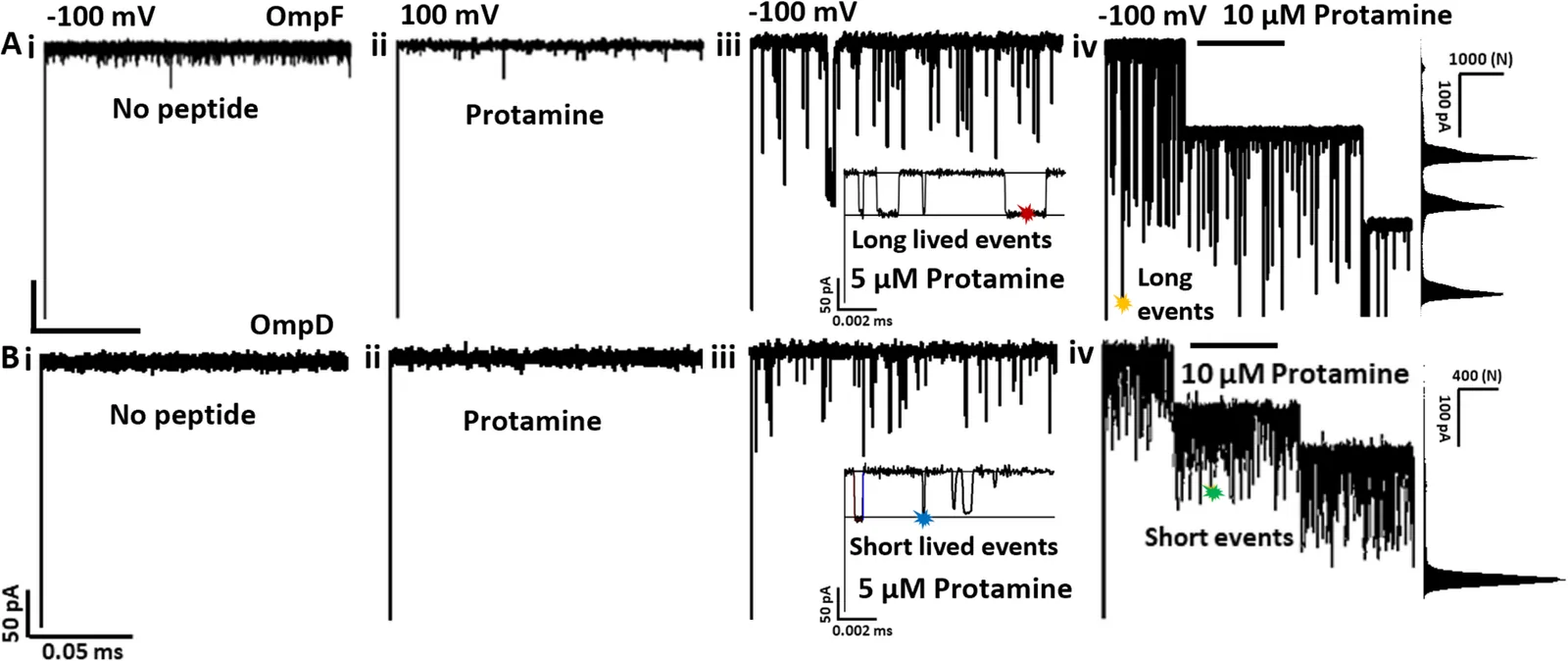
Typical ion current recordings through single trimeric OmpF and OmpD channels reconstituted into planar lipid membranes in the presence of Protamine added to the cis side of the membrane. (**A-B**,** i**) In the absence of peptide at an applied voltage of -100 mV. (**A-B**,** ii**) in the presence of Protamine peptide at applied reversal voltage of 100 mV through OmpF and OmpD channels. (**A-B**,** iii**) In the presence of 5 µM Protamine through OmpF channel showed mixed long and short partial blockage events and through OmpD channel showed short partial blockage events at an applied voltage of -100 mV. Insets show long-lived (red star) and short-lived blockage events (blue star). (**A-B**,** iv**) In the presence of 10 µM Protamine through OmpF and OmpD channel monomers with 3- step complete closure displayed mostly long blockage events (yellow star, OmpF) and short blockage events (green star, OmpD) at an applied voltage of -100 mV. The fully open state of OmpF and OmpD channels was marked with a solid line. On the right-hand side corner panel, the corresponding amplitude histograms were shown. The data is representative of *n* ≤ 10 for OmpF and *n* = 2 for OmpD. Experimental conditions: T = 25 °C, 1 M KCl, 20 mM MES, pH = 6

The concentration dependence of the Protamine interaction with OmpF was investigated. Addition of the large cationic peptide 10 µM protamine (Ptm, with 31 amino acid and 20 arginine residues) to the cis (extracellular) side of the membrane required trans-negative potentials (− 100 mV) to induce interaction with the lumen of the OmpF channel. When Ptm was added to the trans side, voltage dependence was reversed: positive potentials increased event frequency, while negative potentials produced no current (Fig. 1Ai, ii). In the absence of the peptide, current fluctuations through the channel were very stable without any modification in the channel conductance (Fig. 1Ai). No binding events were observed when the electric field was reversed, positive potentials on the trans (intracellular) side, indicating voltage-dependent interaction (Fig. 1Aii). On applying negative potential, the peptide interaction was prominent whereas on reverse potential no binding effect was noticed. At lower concentration of 5 µM Ptm single monomer closure of the channel with mixed long-lived (assigned 1, ≥ 2.5 ms) and short-lived (assigned 0, > 100 µs) blockage events were observed (Fig. 1Aiii, inset). The averaged residence time (τ_*off*_) was ~ 200 µs. Increase in the applied voltage led to an increase of the residence time. At higher transmembrane potential − 100 mV or -150 mV, OmpF channel exhibited gating behavior, characterized by stepwise complete closure of the three monomers, in the presence of higher concentration 10–20 µM of Ptm (Fig. 1Aiv). Larger aqueous concentrations of the peptide led to an increase in the number of peptide-induced blocking events, and this can be visually quantified by a close inspection of the amplitude histogram of such events (Fig. 1A, right corner panel).

No change in the OmpD channel conductance was observed in the absence of peptide (Fig. 1Bi). No blocking events were observed when the electric field was reversed (Fig. 1Bii). Protamine at lower concentration (5 µM) exhibited weaker and shorter partial blockage events with the channel under the same conditions (Fig. 1Biii, inset show mostly short-lived events (assigned 0, 50–100 µs)). At higher concentrations (10 µM), Ptm produced short‑lived blocking events that caused transient pore blockages or complete channel closure (Fig. 1Biv).

The current–voltage relationship of OmpF and OmpD channels at symmetrical 1 mM KCl was shown (Fig. S1D, E). Event frequency and residence time increased with both peptide concentration and applied voltage for OmpF and OmpD channels (Fig. S2A-C), further supporting voltage-dependent binding. The behaviors were consistent with peptide interaction with the channel but did not provide clear indication of peptide entry into the channel or definitive evidence of full translocation. Protamine induced much longer blocking events, and the event frequency was approximately ten‑fold higher than for OmpD, consistent with considerably weaker engagement of the peptide with OmpD.

To provide an initial assessment of how protamine interacts with OmpF and whether specific constriction‑zone residues contribute to this process, two OmpF mutants that were available during the study period were examined. Prior work has shown that residues in the L3 loop (Asp, Glu, Arg, Lys, Tyr) influence the interaction and translocation of AMPs and antibiotics, motivating the use of the double mutant OmpF (D113N–E117Q) (G = 1.5 ± 0.2 ns) and the single mutant OmpF (E117Q) (G = 3.2 ± 0.2 ns) [32, 33]. Recordings with low protamine concentrations showed complete channel blockage in both mutants (Fig. S3A, B), each measured once, while a short cationic peptide (Tetra‑arginine) produced no events in the double mutant, serving as a negative control (Fig. S3C). The data serve as preliminary indication that these L3 loop residues may contribute to protamine binding, generating hypotheses for future, more comprehensive mutational studies.

Additional recordings in 0.1 mM KCl and 100 mM KCl buffer helped in better understanding of protamine interaction with the porins. In 0.1 M KCl, 20 mM MES, pH 6.0 buffer, the OmpF and OmpD channels displayed rapid closure on addition of protamine at -100 mV (Fig. S4). Recordings were reproduced 3 times for 0.1 mM KCl and 1 time for 100 mM KCl.

Indirect methods such as dynamic light scattering, UV spectroscopy and fluorescence spectroscopy were used to perform liposome swelling assay, Zeta potential size measurement and substrate selective supramolecular tandem assay to further examine protamine interaction with OmpF channel (Fig. S5 and S6). POPC liposomes reconstituted with OmpF porins showed changes in the optical density (rate of swelling) and osmolality upon Protamine addition [30] (Fig. S5A), and Zeta- potential measurements indicated only slight size changes (Fig. S5B). Protamine diffusion across OmpF‑containing liposomes was low, and no substantial zeta‑potential differences were observed relative to controls. Fluorescence displacement experiments showed that protamine effectively displaced Lucigenin (LCG) from the P‑sulfonatocalix[4]arene (CX4) host, and liposomes reconstituted with OmpF containing the host-dye complex exhibited corresponding fluorescence changes [34, 35] (Fig. S6Ai, ii).

However, these indirect fluorescence‑displacement and liposome‑swelling assays report secondary changes rather than pore‑level events. These results are exploratory and were not used to draw mechanistic conclusions and instead served only to generate hypotheses and motivate future work in which more definitive transport assays can be incorporated.

**Fig. 2:**
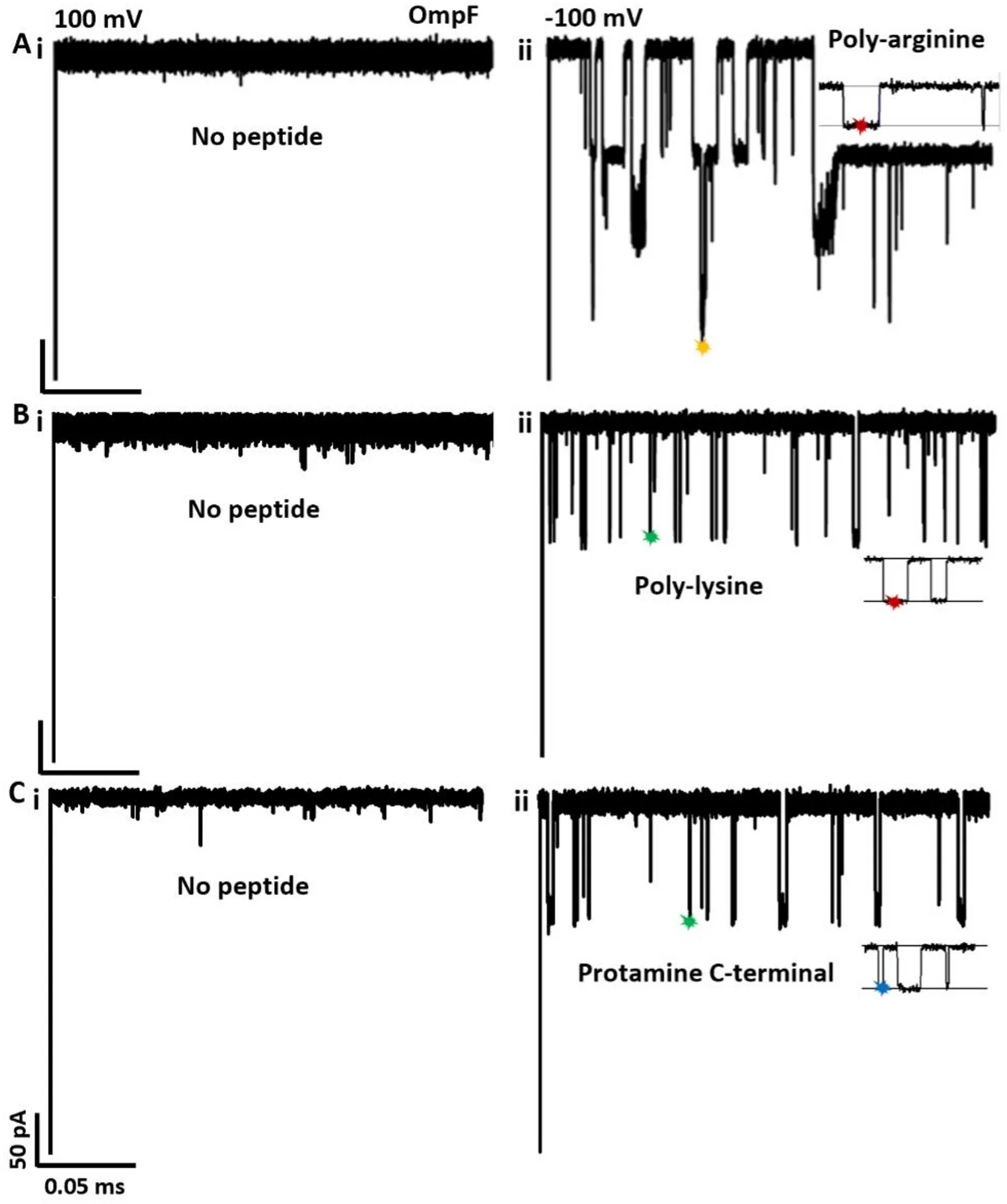
Typical ion current recordings through single trimeric OmpF channel reconstituted into planar lipid membranes in the presence of large cationic peptides added to the cis side of the membrane. (**A-C**,** i**) In the absence of peptides at a reversal applied voltage of 100 mV. (**A**,** ii**) In the presence of 5 µM Poly-arginine showed effective gating of the channel with two-step closure displaying mixed long (yellow star) and short blockage events. (**B**,** ii**) In the presence of 5 µM Poly-lysine displayed mostly short blockage (green star) events. (**C**,** ii**) In the presence of 5 µM Protamine C-terminal end displayed mostly short blockage (green star) events. Insets show mixed long-lived (red star) in case of Poly-arginine and Poly-lysine and short-lived (blue star) for Protamine C-terminal blocking events. Current recordings with peptides were performed at an applied voltage of -100 mV. The data is representative of *n* = 1 for Poly-arginine, *n* = 4 for Poly-lysine and *n* = 2 for Ptm C-terminal end. Experimental conditions: T = 25 °C, 1 M KCl, 20 mM MES, pH = 6

Interaction of longer cationic peptides such as poly‑arginine and poly‑lysine with the OmpF channel was examined under defined experimental conditions. Both peptides induced mixed long and short current‑blockade events, including two‑step closures for poly‑arginine (Fig. 2A, B; insets), indicating pronounced voltage‑dependent interactions similar to those observed for protamine. Poly‑arginine produced long‑lived blockages that frequently involved multiple monomer closures. The number of detectable events and their residence times increased with peptide length. The average *τ* of blockage events at -100 mV applied voltage were ~ > 1.0 ms for poly-arginine, ~ 100 µs for poly-lysine. Additional measurements using the enzymatically generated and purified C‑terminal fragment of protamine (RAGRRRR) (Fig. 2C) showed that this seven‑residue peptide produced larger current amplitudes and longer dwell times (Fig. S3D), comparable to poly‑lysine and other short cationic peptides, consistent with detectable monomer‑level interactions with OmpF.

Protamine produced complete OmpF channel closures at − 100 mV in 1 M KCl, 20 mM MES, pH 6, demonstrating strong voltage‑dependent engagement under these conditions.

### Voltage‑Dependent Transient Interactions of Short Cationic Peptides with the OmpF

**Fig. 3:**
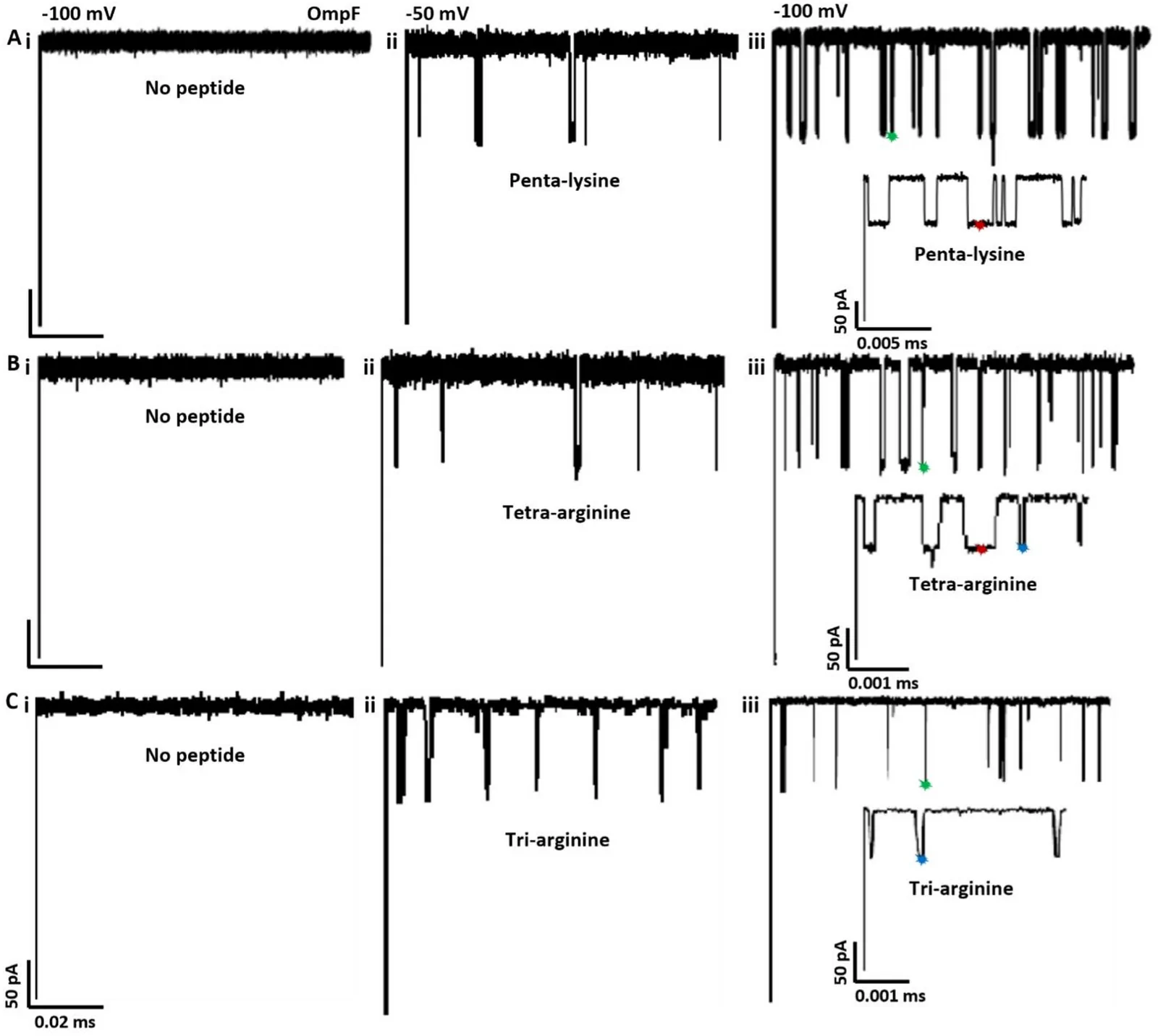
Typical ion current recordings through single trimeric OmpF channel reconstituted into planar lipid membranes in the presence of short cationic peptides added to the cis side of the bilayer membranes. (**A-C**,** i**) In the absence of peptides at an applied voltage of -100 mV through OmpF channel. (**A-C**,** ii**) 15 µM of Penta-lysine, 3 µM of Tetra-arginine, and 10 µM of Tri-arginine displayed short blockage events at an applied voltage of -50 mV. (**A-C**,** iii**) Penta-lysine and Tetra-arginine displayed mixed long-and short-lived blockage events (red and blue star) while Tri-arginine displayed mostly short-lived blockage events (blue star) at an applied voltage of -100 mV. Insets show single monomer blockage of OmpF channel through short blocking events (green star). The data is representative of *n* = 10 for Penta-lysine, *n* = 3 for Tetra-arginine and *n* = 2 for Tri-arginine. Experimental conditions: T = 25 °C, 1 M KCl, 20 mM MES, pH = 6

Single‑channel recordings were used to characterize how short cationic peptides interact with OmpF. When peptides were added to the cis side, negative potentials increased the frequency of detectable current blockages, whereas positive potentials produced no events (Fig. 3A–Cii). At − 50 mV and − 100 mV, penta‑lysine, tetra‑arginine, and tri‑arginine all produced reversible current interruptions (Fig. 3). These appeared as transient fluctuations consistent with short‑lived peptide encounters with the pore. Penta‑lysine and tetra‑arginine produced complete monomer‑level closures, while tri‑arginine generated partial blockages (Fig. 3A–Ciii). Event frequency increased with voltage, indicating that the applied field modulates encounter probability. Fluorescence displacement assays showed that peptides could displace the CX4–LCG reporter from OmpF‑containing liposomes (Fig. S6B–Ci, ii), supporting the occurrence of peptide–pore interactions without providing structural information.

**Fig. 4:**
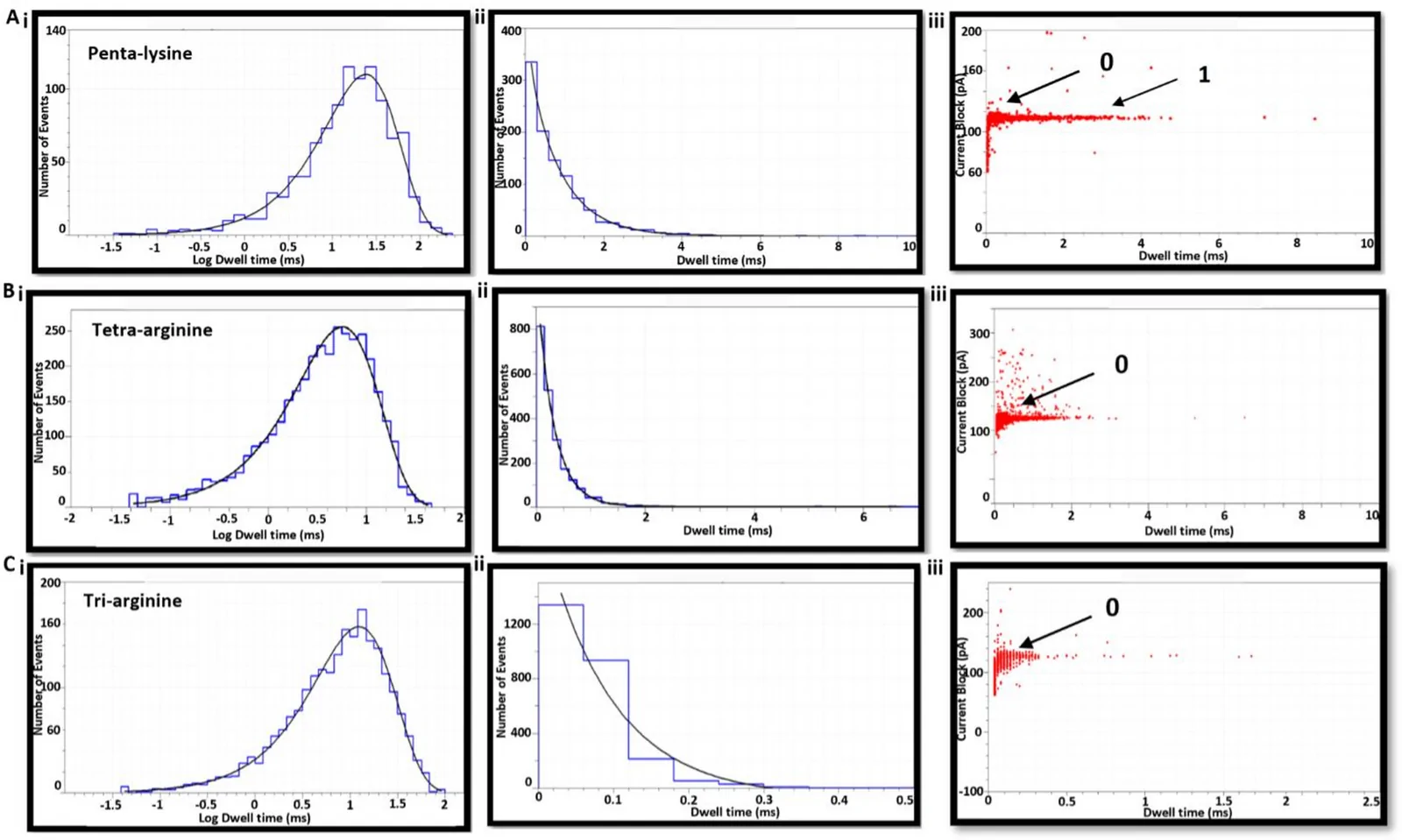
Semilogarithmic histogram and scatter plot of short cationic polypeptides interaction with OmpF channel reconstituted into planar lipid membranes. (**A-C**,** i**) Semilogarithmic histogram of inter-event intervals (*ν*_*m*_) of Penta-lysine (*τ*_*on*_ = 29.87 ± 1.19 ms), Tetra-arginine (*τ*_*on*_ = 5.78 ± 0.11 ms) and Tri-arginine (*τ*_*on*_ = 12.41 ± 0.02 ms). The distribution was fitted using a single Gaussian function (solid line) with a fixed baseline powered by the Levenberg-Marquardt algorithm using Origin to determine the open-state lifetime where *R²* > 0.95. (**A-C**,** ii**) Dwell-time histogram of Penta-lysine (*τ*_*off*−0_ = 0.10 ± 0.20 ms, *τ*_*off*−1_ = 0.83 ± 0.04 ms), Tetra-arginine (*τ*_*off*−0_ = 0.28 ± 0.05 ms, *τ*_*off*−1_ = 0.57 ± 0.45 ms), Tri -arginine (*τ*_*off*−1_ = 0.09 ± 0.02 ms) fitted with single exponential decay fit to determine the close-state lifetime. The solid lines represent the fit where *R*^*2*^ > 0.94. (**A-C**,** iii**) Scatter plot of current block amplitudes versus dwell time of peptides added to the cis side of the membranes at an applied voltage of -100 mV. The plots are representative of Penta-lysine, Tetra-arginine and Tri-arginine from pooled data mentioned earlier. Experimental conditions: T = 25 °C, 1 M KCl, 20 mM MES, pH = 6

Two general classes of events were observed at − 100 mV: short‑lived (*τ*_*off*−0_) and longer (*τ*_*off−1*_). Short‑lived events were voltage‑independent and attributed to brief collisions at the pore entrance; they were excluded from kinetic estimates because they do not reflect sustained pore occupancy [22, 28]. Long‑lived events increased in frequency with voltage and peptide length (Fig. 4). These longer events represent more prolonged interactions but do not indicate deeper penetration or transport.

Dwell‑time histograms were fitted with exponential models to describe the distributions. Penta‑lysine and tetra‑arginine required two‑exponential fit, while tri‑arginine was described by a single exponential fit. The extracted values: penta-lysine (*τ*_*off*−0_ = 0.10 ± 0.20 ms and *τ*_*off−1*_ = 0.83 ± 0.04 ms), tetra-arginine (*τ*_*off*−0_ = 0.28 ± 0.05 ms and *τ*_*off−1*_ = 0.57 ± 0.45 ms) and tri-arginine (*τ*_*off−1*_ = 0.09 ± 0.02 ms) reflect the durations of resolvable events under the 10‑kHz filter limit. These values are descriptive estimates of event durations rather than experimentally validated kinetic constants. Longer peptides produced longer average dwell times (> 100 µs for penta‑lysine and tetra‑arginine; ≤50 µs for tri‑arginine) (Fig. 4A-Bii, iii), illustrating how peptide size influences the detectability of interaction events.

**Fig. 5:**
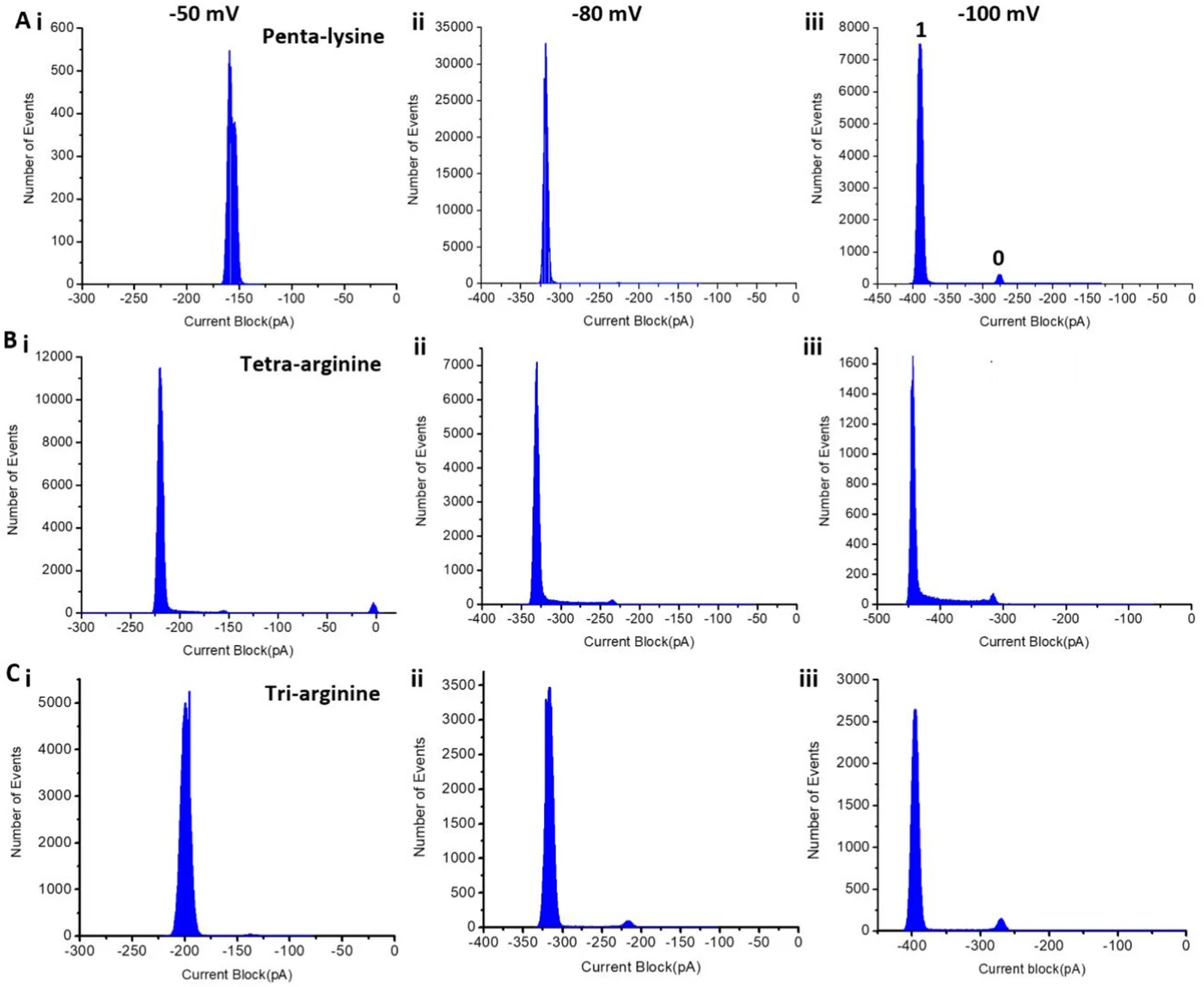
Typical amplitude histograms displaying voltage dependent transient single-channel blockage by short cationic peptides of the single trimeric OmpF channel reconstituted into planar lipid membranes. (**A**) In the presence of 15 µM Penta-lysine, (**B**) In the presence of 3 µM Tetra-arginine and (**C**) In the presence of 10 µM Tri-arginine. Peptides were added to the cis side of the membranes and the applied voltage were − 50 mV (**i**), -80 mV (**ii**) and − 100 mV (**iii**). At − 100 mV all the three short peptides showed major and minor peaks (0). The plots are representative of Penta-lysine, Tetra-arginine and Tri-arginine from pooled data. Experimental conditions: T = 25 °C, 1 M KCl, 20 mM MES, pH = 6

Amplitude histograms revealed two recurring blockade populations: peak 0 (low‑amplitude, short‑duration) and peak 1 (higher‑amplitude, longer‑duration) (Fig. 5). Peak 0 occurred across all voltages, while peak 1 increased with peptide length and voltage. Peak 1 amplitudes were consistent with more extensive pore occupancy [28] but do not confirm insertion depth or specific binding sites. The frequency of peak 1 events at − 100 mV followed the order tetra‑arginine (200–250) > tri‑arginine (150–200) > penta‑lysine (100–150) (Fig. 4A-Ci), reflecting differences in detectable event patterns rather than mechanistic binding strength. Peak 0 frequencies at -100 mV were approximately: protamine (> 3000), poly‑arginine (> 5000), poly‑lysine (~ 2000), protamine C‑terminal fragment (~ 500), penta‑lysine (~ 1500) for, tetra‑arginine (~ 500), and tri‑arginine (~ 800). For OmpD, protamine‑produced ~ 100–300 events. These values represent the detectable subset of resolvable blockades.

Event‑distribution plots illustrated typical blockage patterns. Detection thresholds were set at 3–4× the baseline RMS noise, with continuous baseline monitoring and drift correction. Events were rejected when ambiguous, overlapping, or occurring during bilayer instability. Higher voltages (150–200 mV) increased intrinsic OmpF gating, so lower potentials (50–100 mV) were used to isolate peptide‑specific blockages.

Tri‑arginine produced only short‑lived events in classical bilayers, highlighting the limitations of the setup for fast interactions. A chip‑based automated patch‑clamp system was therefore used to improve temporal resolution (see Methods, 33).

**Fig. 6:**
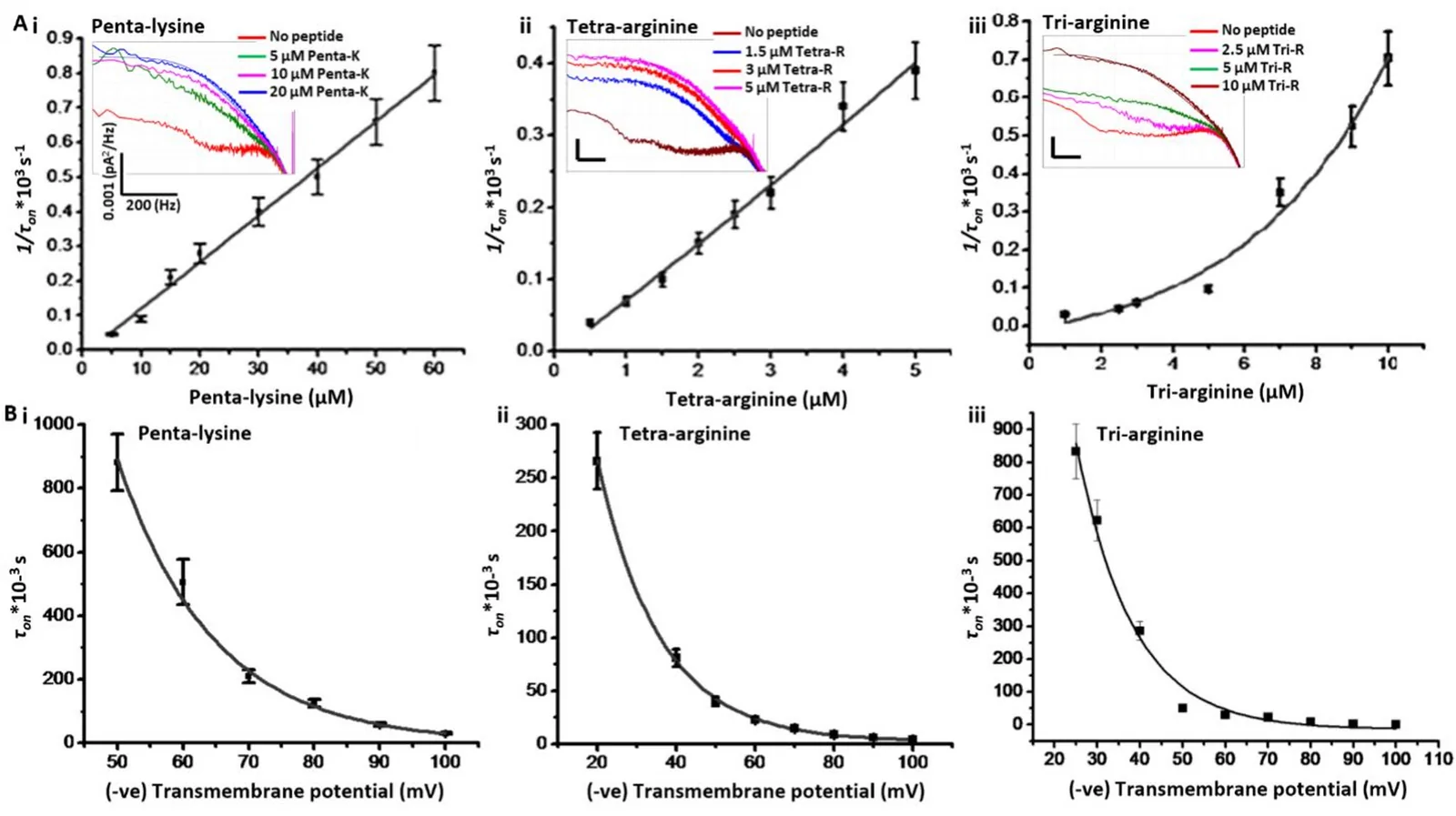
Concentration dependence of rate constant (*k*_*on*_) and voltage dependence of inter-event interval (*τ*_*on*_) of short cationic peptides. (**A**) Concentration-dependence of association rate constant (*k*_*on*_) of peptides Penta-lysine (**i**), Tetra-arginine (**ii**) and Tri-arginine (**iii**) measured at -100 mV fitted using single exponential growth fit yielding mean *k*_*on*_ with SEM. Insets show one -sided power spectral densities of the noise in the current through a single fully open OmpF channel in the presence of cationic peptides at an applied voltage of -100 mV exceed by ~ 2 orders of magnitude. The cationic peptides produced measurable noise which increased with increase in concentration in comparison to 1 M KCl buffer. Smooth solid lines through the spectra are Lorentzian fit powered by the Levenberg-Marquardt algorithm in Origin showing suppression yielding mean current amplitude *R*^*2*^ > 0.95. Log Scale bar (x-label = Spectral density, y-label = Frequency). (**B**) Voltage-dependence of inter- event interval time (*τ*_*on*_) of peptides Penta-lysine (**i**), Tetra-arginine (**ii**) and Tri-arginine (**iii)** measured at -100 mV. 5 µM of peptides were added to the cis side of the bilayer membranes. The data was an average of ~ 3 individual measurements of each peptide fitted using single exponential decay fit yielding mean *τ*_*on*_ with SEM. The solid lines represent the fit where *R*^*2*^ > 0.95. The pooled data is mentioned in the results text. Experimental conditions: T = 25 °C, 1 M KCl, 20 mM MES, pH = 6

Power-spectrum analysis [36, 37] revealed characteristic times of ~ 500 µs for penta‑lysine, ~ 300 µs for tetra‑arginine, and ~ 30 µs for tri‑arginine (Fig. 6A; insets). Addition of peptides generated excess noise described by single Lorentzians. At -100 mV, the low-frequency noise exceeded that of the channel in pure salt solution by two orders of magnitude. Earlier studies showed that such noise could be used to estimate transport parameters [27]. Here, the values indicate microsecond‑scale interactions but should be viewed as preliminary descriptors rather than definitive kinetic parameters. The spectra suggest interactions near the narrow region of the pore, but do not resolve structural modes.

Increasing peptide concentration decreased the mean inter‑event interval (*τ*_*on*_) indicating more frequent detectable encounters (Fig. 6A). Turn-off times (*τ*_*off*_) were largely concentration‑independent. These trends are consistent with encounter-driven behaviour but do not define a mechanistic kinetic scheme.

**Fig. 7:**
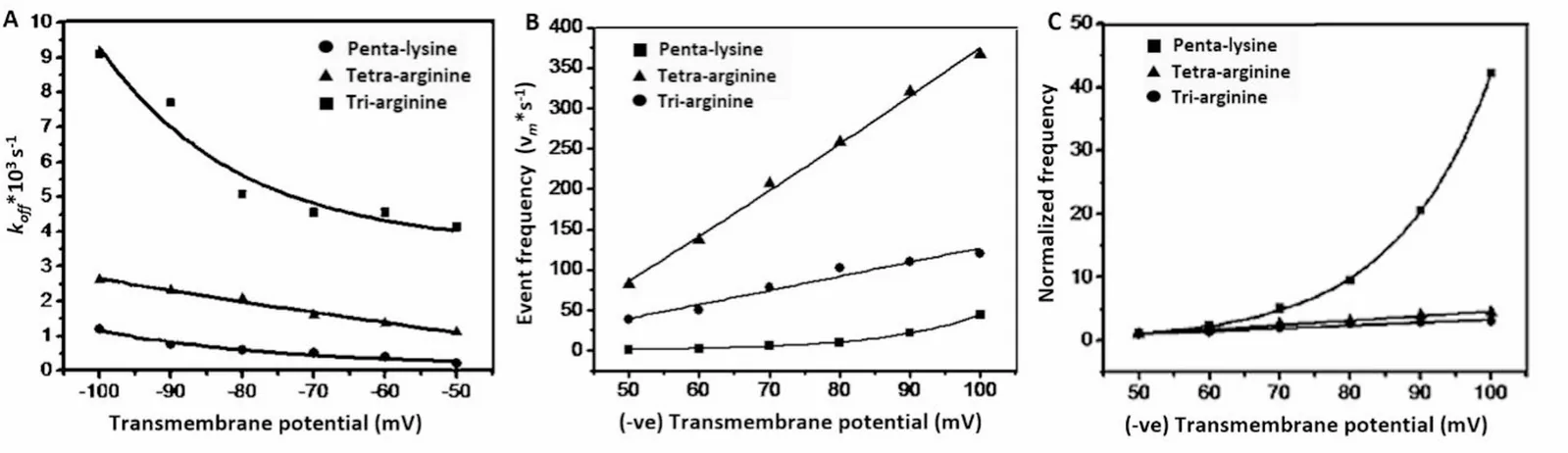
Voltage-dependence of (*k*_*off*_) and event frequency (*ν*_*m*_) of short cationic peptides. (**A**) Voltage-dependence of dissociation rate constant (*k*_*off*_) of peptides Penta-lysine, Tetra-arginine and Tri-arginine measured at -100 mV. (**B**) Voltage-dependence of event frequency of the major peak 1 measured at -100 mV. (**C**) Normalized event frequency at -50 mV increased with applied voltage. 5 µM of peptides were added to the cis side of the bilayer membranes. The data fitted using single exponential growth fit yielding mean *k*_*off*_. The solid lines represented the fit where *R*^*2*^ > 0.94. Experimental conditions: T = 25 °C, 1 M KCl, 20 mM MES, pH = 6

Voltage strongly influenced interaction patterns due to peptide charge [38, 39]. Higher voltages increased event frequency and altered dwell‑time distributions (Fig. 7A-C). These effects reflect voltage‑enhanced encounter probability and sustained pore occupancy, not electrophoretic transport. The association (*k*_*on*_) and dissociation (*k*_*off*_) values were derived from *τ*_*on*_ and *τ*_*off*_ using standard approximations [36, 39, 40] and should be interpreted as descriptive estimates. Comparisons of cis‑ and trans‑side addition at -100 mV (Table 1) showed similar *k*_*off*_ values, indicating that direction of addition did not substantially alter detectable interaction patterns under equivalent electrical conditions.

**Table 1:** Table displaying kinetics of cationic peptides interacting with OmpF

Concentration of peptides: 5 µM	*kon* = 1/*τon*[c] or ν/3[c] (Ms)^-1^ *10^-6^ (Cis side, -100 mV)	*kon* = 1/*τon*[c] (Ms)^-1^ *10^-6^ (Trans side, 100 mV)	*koff* = 1/*τ* (s^-1^)*10^-3^	*Kf = kon/koff* (M^-1^)	П (Partition coefficient) = *Kf*[pept]^*^
Protamine	91	104	3	27829	5983
Poly-arginine	50	33	14	35211	7570
Poly-lysine	16	7	2	11225	2413
Penta-lysine	9	10	1	10580	2275
Tetra-arginine	79	100	2.0	4506	969
Tri-arginine	20	49	10	1951	419

Binding dependence wasbased on peptide molecular mass and peptide length, [pept]* = 0.215 M. The datawas obtained by taking average of 5 individual measurements of Protamine and 3 individualmeasurements of large and short each peptide at T = 25°C and in 1 M KCl, 20 mMMES, pH = 6

In summary, this exploratory study describes voltage‑dependent current‑blockade phenomena produced by cationic peptides interacting with OmpF under defined experimental conditions. Peptide length, concentration, and applied potential influenced the frequency and duration of detectable events. Automated patch‑clamp recordings enabled detection of rapid blockades not resolvable in classical bilayers. The kinetic parameters derived from *τ*_*on*_ and *τ*_*off*_ serve as preliminary descriptive estimates of encounter dynamics and should not be interpreted as mechanistic constants or evidence of transport. Overall, the results characterize how short cationic peptides transiently occupy the OmpF pore under an applied electric field, without demonstrating translocation or mechanistic specificity.

## Discussion

The recordings provide clear qualitative evidence of voltage‑dependent interactions between cationic peptides and the porins OmpF and OmpD. Protamine (Ptm) produced reproducible current blockages in both channels, with pronounced events under trans‑negative potentials of ~ 100–150 mV. These signatures indicate detectable peptide–pore engagement but do not demonstrate peptide passage through either porin. As noted previously, the gating behavior observed here and elsewhere [41] is consistent with interactions at or near the channel entrance that lead to transient or complete closures rather than translocation. Thus, the Ptm‑induced blockages reflect reversible pore occupancy rather than permeation.

Channel‑specific differences were evident: OmpF exhibited longer and more frequent Ptm‑induced blockages than OmpD, consistent with prior work showing that constriction‑zone electrostatics modulate peptide recognition [39, 42, 43].

Longer cationic peptides such as poly‑arginine and poly‑lysine produced mixed blockade events without evidence of transport [44, 45]. Comparable patterns have been reported across biological nanopores, including α‑hemolysin [46, 47, 22, 48–50, 30, 51], where cationic and flexible peptides often generate transient or heterogeneous blockages that reflect pore engagement rather than confirmed translocation.

Smaller cationic peptides including the protamine C‑terminal fragment, penta‑lysine, tetra‑arginine, and tri‑arginine showed voltage‑ and length‑dependent interaction patterns [52]. Although hydrophobicity and conformational flexibility are known to influence peptide–pore interactions [27, 39, 53, 54], the present experiments were not designed to isolate these factors, and such interpretations remain speculative.

A key observation is that residence times increased with voltage. While compatible with stronger electrostatic engagement, this trend does not support electrophoretic translocation. Instead, higher voltages appear to increase the likelihood and duration of reversible pore occupancy events, consistent with earlier nanopore studies reporting voltage‑enhanced interactions without transport [44, 52]. The uniformity of current blockages suggests reproducible interaction behavior, but the experiments cannot resolve conformational states or structural transitions within the pore.

Although structural or computational analyses were not performed here, recent molecular dynamics simulations highlight the importance of electrostatic steering [55] and transient binding poses that do not necessarily lead to permeation [50, 56–59]. These studies complement but do not resolve the pore‑level behaviors observed experimentally.

Future work should focus on approaches capable of distinguishing binding, gating, and permeation mechanisms. Targeted porin mutagenesis could clarify how constriction‑zone electrostatics shape peptide recognition, while time‑resolved structural methods could identify peptide positions within the pore. High‑field molecular dynamics simulations may reveal voltage‑dependent binding intermediates, and single‑channel kinetic modeling could separate gating from partial insertion events. Comparative nanopore assays using pores with established transport profiles would further help benchmark event signatures against confirmed translocation. Together, these strategies would enable mechanistic resolution beyond what is accessible from single‑channel recordings alone.

### Study Limitations

The study did not include error bars, confidence intervals, or statistical testing because the experimental design focused on qualitative characterization of interaction patterns rather than quantitative kinetic comparison. The traces and dwell‑time distributions therefore represent typical behaviors under the tested conditions. During the study period, it was not possible to expand the experimental dataset, obtain additional mutants and replicates or perform further controls and statistical analyses required for meaningful quantitative comparison. The mechanistic conclusions remain preliminary, and the scientific advance is necessarily incremental. As a result, the current dataset could not support statistically grounded conclusions regarding peptide‑specific interaction behavior with future work incorporating adequate replication and statistical evaluation.

Several limitations were considered. The experiments did not include neutral peptide controls or assays to exclude aggregation or bilayer perturbation [60] and the artificial bilayer system lacks the complexity of the native outer membrane. Thus, the observed behaviors reflect intrinsic OmpF properties rather than full physiological transport. Differences in peptide length and charge [61] likely contribute to the distinct interaction patterns observed, but their relative contributions could not be fully resolved from the available data. Future work incorporating neutral peptides, porin‑targeted molecules, in vivo validation, and a broader porin panel including Omp‑Pst1 from *P.stuartii* [55] and OprF from *P.aeruginosa* [62] would help clarify the determinants of cationic peptide-porin interactions.

Limited preliminary observations of two OmpF variants (E117A and R312A) were intended to highlight that potential negatively charged residues in the L3 loop may influence peptide interaction; however, the available mutants were insufficient to draw firm conclusions about residue‑specific contributions requiring extensive mutational studies. Similar observations in antibiotic–porin studies [42, 63, 64] indicated that charged residues can modulate ligand interaction patterns, but in the present context these comparisons should be viewed as contextual rather than mechanistic. Together, these external studies reinforce the interpretation that the voltage‑dependent blockages observed reflect transient peptide–pore interactions and electrostatic engagement rather than definitive evidence of peptide translocation [65]. Given the limited availability of mutants, the absence of replication, and the inability to perform systematic electrostatic perturbations or control experiments, these observations cannot support quantitative or residue‑level mechanistic conclusions.

Indirect assays reported indirect changes and therefore did not allow definitive attribution of the observed signals to specific binding or translocation mechanisms. Their sensitivity to liposome size variability, membrane heterogeneity, and procedural nuances limits the ability to attribute the observed signals to specific binding or transport mechanisms.

The 10‑kHz low‑pass filter limits detection to events longer than ~ 50–100 µs, meaning very fast transient interactions may not be captured. Accordingly, the reported dwell‑time distributions reflected only the resolvable subset of peptide‑induced events. The instrumental cutoff introduced uncertainty into the kinetic interpretation of the data. Because many of the observed events fell close to this threshold, it was likely that fast events were undercounted, residence times may be skewed toward longer values, and overall event frequency and kinetic analyses may be biased. The magnitude of this bias could not be quantified with the presented dataset. It was not possible to implement higher bandwidth recording conditions that would allow reliable detection of sub 50 µs events. Because the experiments were not optimized for quantitative kinetics and many rapid events fell below detection, the kinetic values represent resolution‑limited, preliminary descriptors, not definitive rate constants. They should be interpreted as qualitative indicators of observable trends within the constraints of the system.

Across all experiments, the current traces did not show signatures consistent with peptide passage to the trans side. All events were reversible and compatible with transient pore occupancy followed by return to the cis side. The data therefore reflected interaction, not transport, under the tested conditions. Statements implying deeper penetration or movement through the pore could not be supported by the available evidence. The analysis was exploratory, with future work required to develop models that more accurately reflect the underlying biophysical processes and use improved temporal resolution to accurately characterize fast protamine-porin interactions.

To summarize the limitations the study was constrained by limited biological and technical replication across several experimental conditions, the absence of statistical analysis, and the lack of neutral or scrambled peptide controls needed to exclude nonspecific effects. Mutant coverage was minimal, preventing residue‑level interpretation, and indirect assays could not distinguish adsorption from transport. The temporal resolution of the recording system restricted detection to relatively slow events, biasing kinetic measurements and limiting mechanistic insight. Together with the inability to demonstrate peptide translocation or identify specific binding sites, these factors mean that the findings should be interpreted as qualitative descriptors of interaction behavior rather than statistically or mechanistically definitive conclusions.

## Conclusions

A principal contribution of this work is the establishment of a descriptive electrophysiological dataset and an experimental framework for probing peptide–porin interactions. Cationic peptides produced reversible, voltage‑dependent current blockages in OmpF, reflecting transient pore interactions without evidence of translocation. These observations provide a qualitative description of how short peptides engage with the OmpF lumen and should be interpreted as preliminary rather than explanatory. OmpF functioned as a stable single‑molecule platform, enabling detection of short‑lived, voltage‑modulated events influenced by peptide length and concentration. The blockades represent reversible occupancy followed by return to the cis side, indicating interaction rather than passage across the membrane. Overall, the study offers a foundational experimental framework for characterizing peptide–porin interactions and supports future efforts to examine how antimicrobial peptides engage with bacterial outer membrane channels.

## Supplementary Information

Below is the link to the electronic supplementary material.

Supplementary Material 1

## Data Availability

All data supporting the findings of this study are available within the paper and its Supplementary Information.
