# Supplementary material for: Probing Voltage Dependence Interaction of Cationic Peptides with Bacterial Porins at A Single-Molecule Level
Source: Cell Biochem Biophys. 2026 Aug 14;84(3):4583–97. doi: 10.1007/s12013-026-02148-3 (PMC13585899; doi:10.1007/s12013-026-02148-3)
Supplement: Supplementary file 1 [file 12013_2026_2148_MOESM1_ESM.pdf]

SUPPLEMENTARY INFORMATION

Probing voltage dependence interaction of cationic peptides with bacterial porins at a single-molecule level

Sonal Prasad[1, 2]

[1] Department of Life Sciences and Chemistry, Jacobs University Bremen, Campus Ring 1, 28759, Bremen, Germany.

[2] Present address: Department of Biomedicine and Clinical Sciences, Linköping University, SE-581 83 Linköping, Sweden

Running title: Cationic peptide–porin interactions

Supplementary data contains:

-Supplementary methods text

-Figures with captions (S1 to S6)

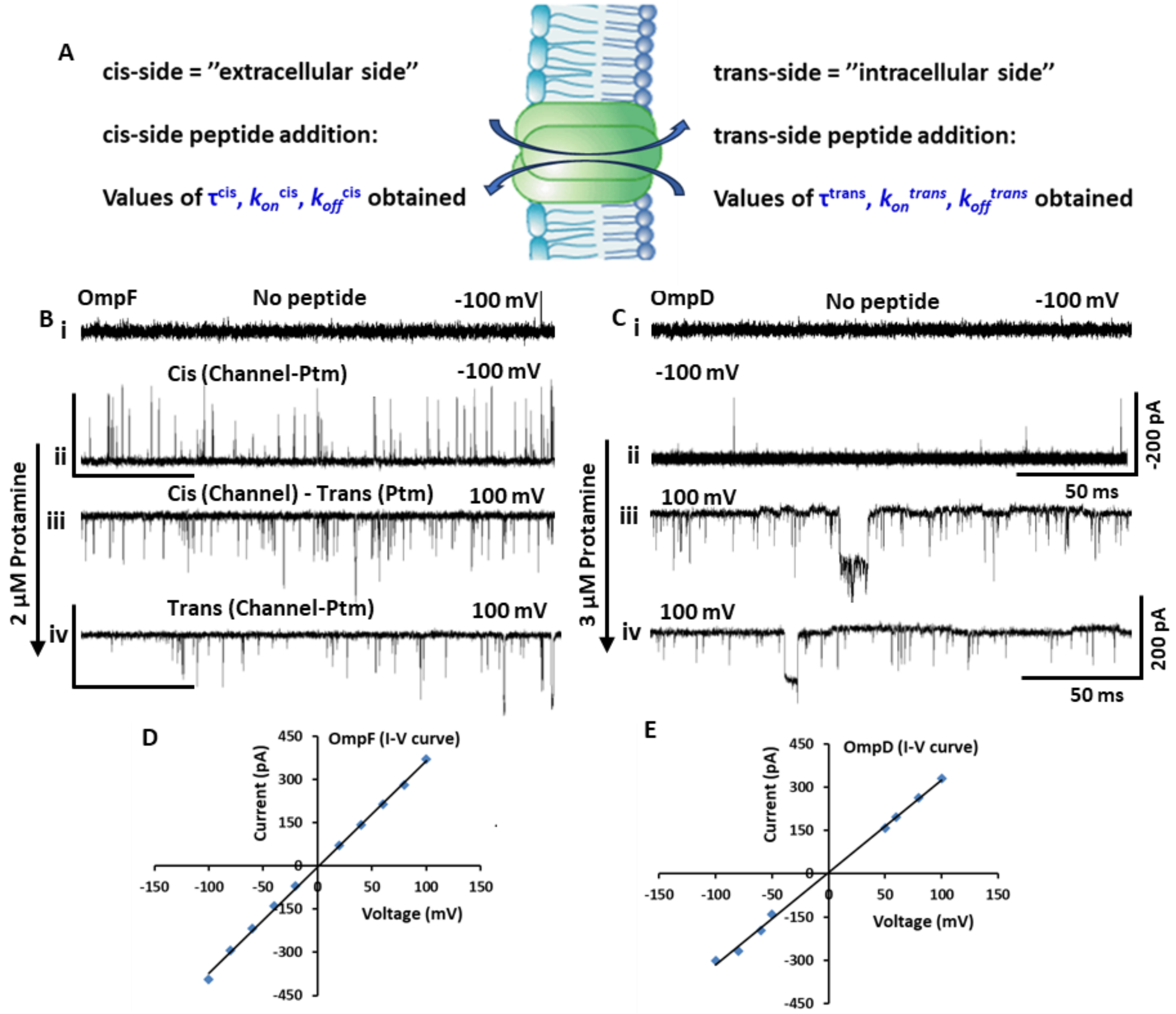

Figure S1. Typical current recordings through OmpF and OmpD channels with Protamine. (**A**) Chemical affinity constants were obtained experimentally to study the affinity of peptides with channels. Cis-side was considered extracellular side of the bilayer membrane where peptides were added and trans-side was the intracellular side of the membrane representation of inside of a cell. (**B-C, i**) In the absence of Protamine at a transmembrane potential of -100 mV through single trimeric OmpF and OmpD channels reconstituted into planar lipid membranes. (**B-C, ii**) In the presence of low concentration Protamine strong (OmpF) and minimal interaction (OmpD) seen at applied potential of -100 mV when peptide and channel added on the cis side of the bilayer membrane. (**B-C, iii**) In the presence of Protamine strong interaction seen with channels at applied potential of -100 mV when peptide added to trans and channel added on the cis side of the bilayer membrane. (**B-C, iv**) In the presence of Protamine weak interaction seen with channels at applied potential of -100 mV when peptide and channel added on the trans side of the bilayer membrane. (**D, E**) Typical I-V curve of the OmpF and OmpD channels showing maximum current of 400 pA at a gating voltage of 100 mV. Experimental conditions: T = 25°C, 1 M KCl, 20 mM MES, pH = 6.

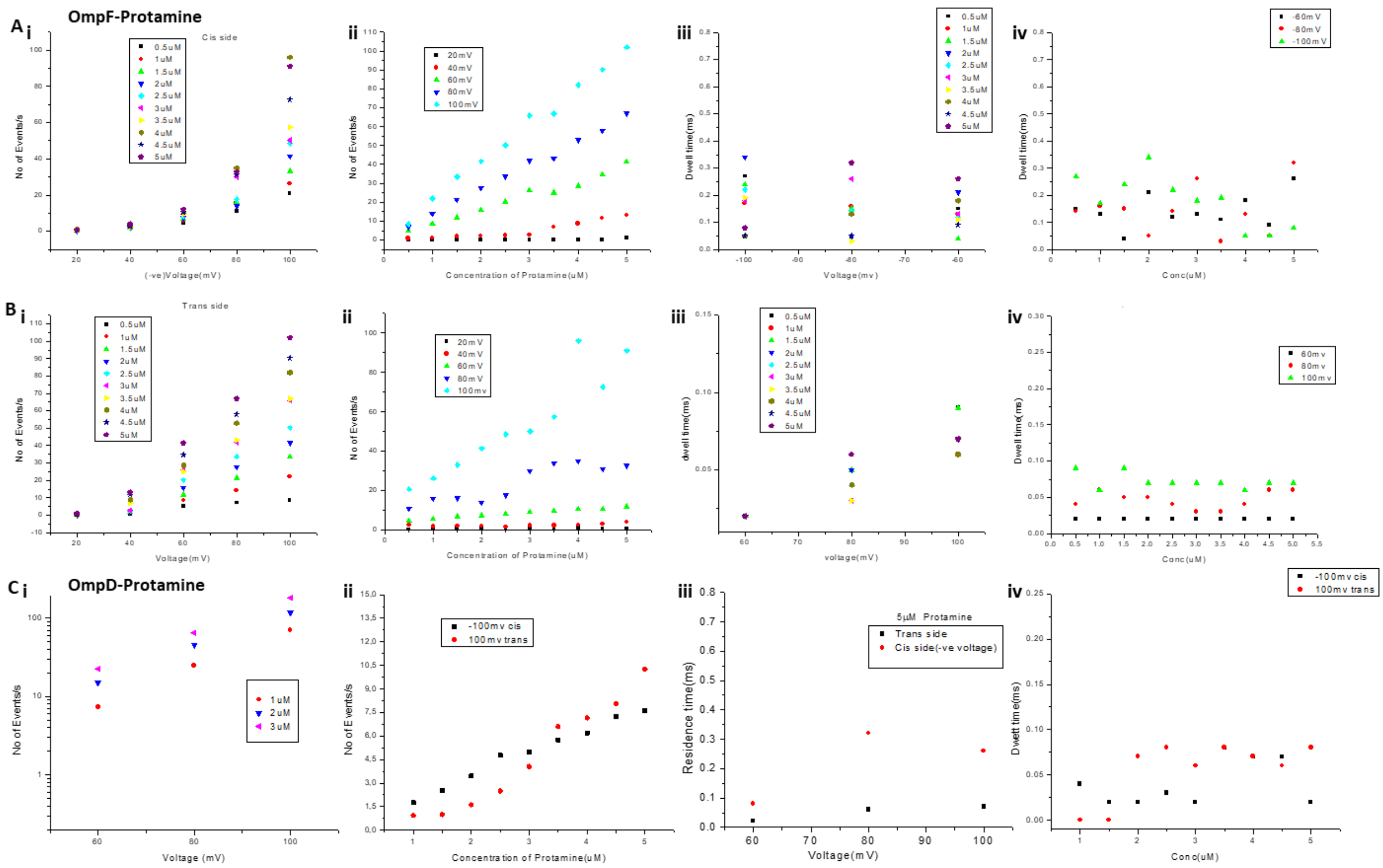

Figure S2. Concentration dependence of event frequency and voltage dependence of dwell time of Protamine through OmpF and OmpD porins. (**A, B**) Event frequency (**i, ii**) and residence time (**iii, iv**) increased with both peptide concentration and applied voltage for OmpF channel after Protamine added to the cis and trans side. (**C**) Event frequency (**i, ii**) and residence time (**iii, iv**) increased with both peptide concentration and applied voltage for OmpD channel after Protamine added to the cis and trans side. Experimental conditions: T = 25°C, 1 M KCl, 20 mM MES, pH = 6.

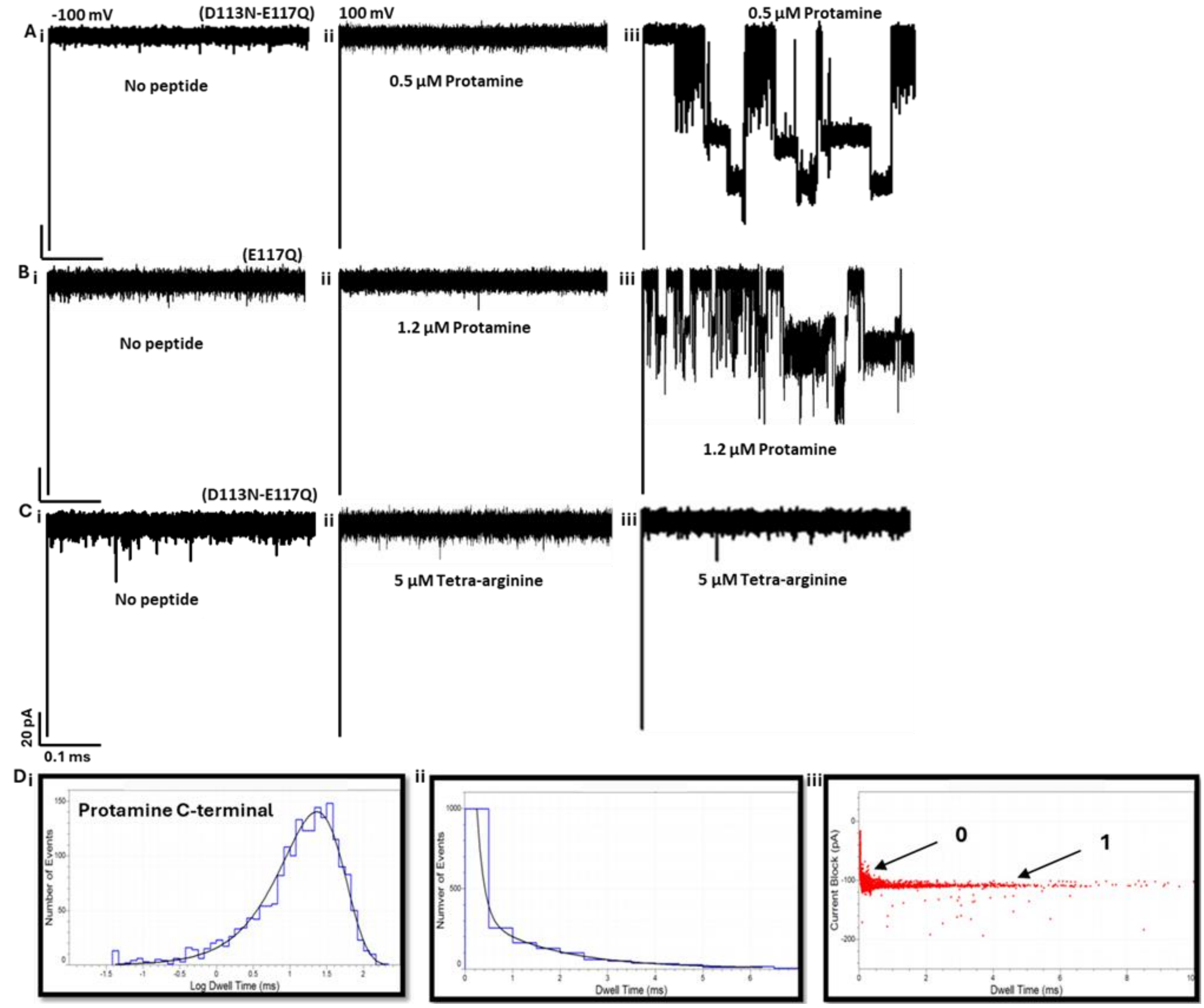

Figure S3. Typical ion current recordings through OmpF mutant porin with Protamine added to the cis side. (**A-C, i**) In the absence of peptides at a transmembrane potential of -100 mV through single trimeric OmpF mutant channels reconstituted into planar lipid membranes (**i**). (**A-C, ii**) In the presence of peptides showing no interaction at a reversal transmembrane potential of 100 mV. (**A-B, iii**) Low concentration of protamine displaying inhibition of the double mutant and single mutant OmpF channels through two-step closure short blockage events at -100 mV. (**C, iii**) Tetra-arginine displayed no interaction with the double mutant OmpF channel suggesting specificity of Protamine interaction with OmpF porin. (**D**) Semilogarithmic histogram of inter-event intervals (**i**) of Protamine C-terminal end (τ_{on} = 29.87 ± 1.19ms), dwell-time histogram (**ii**) ($\tau_{off\text{-}0}$ = 0.10 ± 0.20 ms, $\tau_{off\text{-}1}$ = 0.83 ± 0.04 ms) and scatter plot of current block amplitude versus dwell time (**iii**) at an applied voltage of -100mV. Experimental conditions: T = 25°C, 1 M KCl, 20 mM MES, pH = 6.

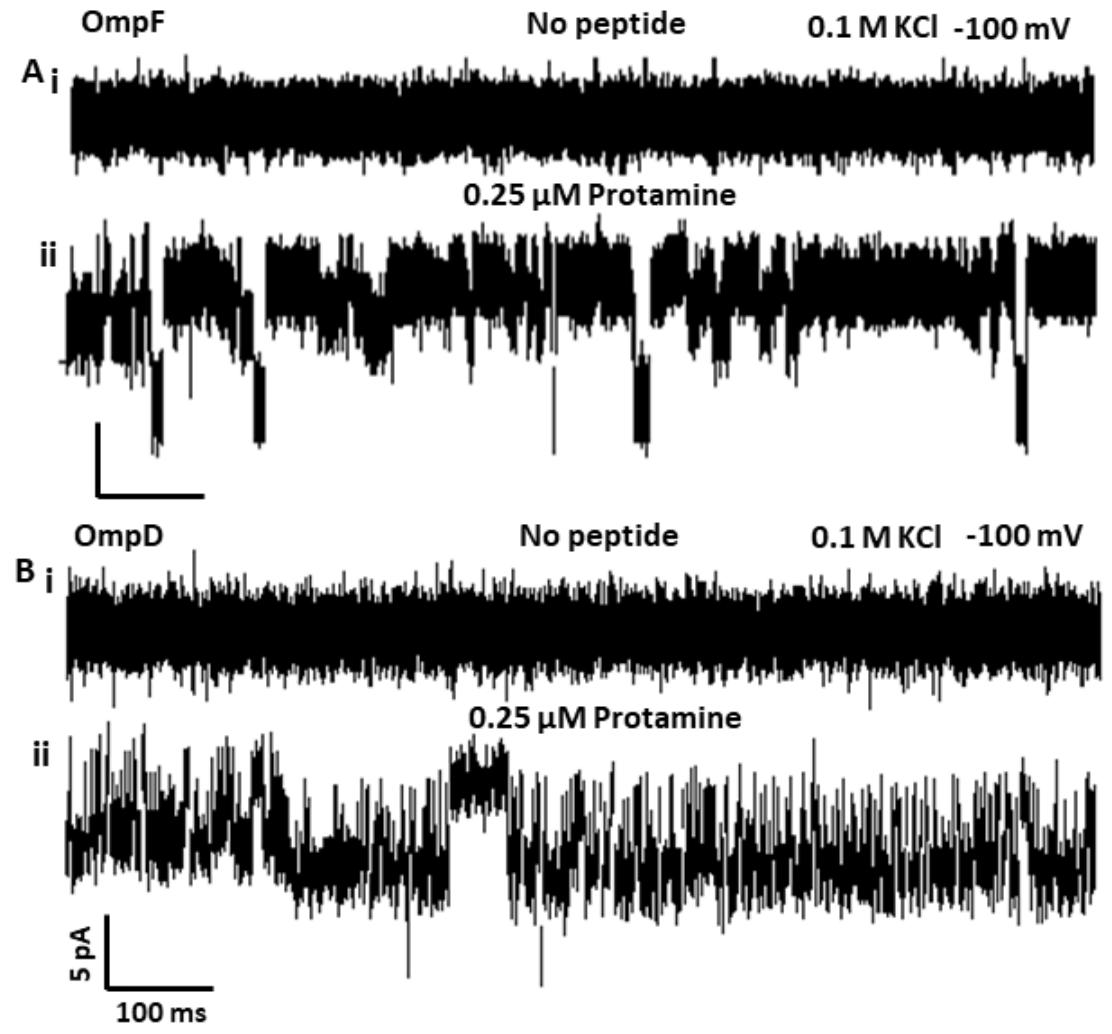

Figure S4. Typical ion current recordings through OmpF and OmpD with Protamine added to the cis side in 0.1 M KCl. (**A-B, i**) In the absence of Protamine at a transmembrane potential of -100 mV through OmpF and OmpD channels. (**A-B, ii**) In the presence of low concertation 0.25 μM Protamine showed minimal interaction with OmpF and OmpD at -100 mV in 0.1 M KCl, 20 mM MES, pH 6.0.

Liposome swelling assay

OmpF porin (50 ng/µl in 1% octyl-POE) was reconstituted into multilamellar liposomes following the procedure of Nikaido and Rosenberg (1). Liposomes were prepared from Escherichia coli total lipid extract (phosphatidylethanolamine, phosphatidylglycerol, and cardiolipin; 2 mg/ml) in the presence of 15% (w/v) dextran (molecular weight 40,000) to ensure vesicle entrapment. Size distributions were determined using a Nano-ZS ZEN3600 Zetasizer (Malvern Instruments). Control liposomes were generated in parallel without addition of porin.

Osmolarity of all test solutions was measured using an Osmomat 30 osmometer (Gonotec). For swelling assays, 30 µl of liposome or proteoliposome suspension was diluted into 630 µL of isotonic test solution prepared in 5 mM Tris-phosphate buffer, pH 7.4, within a 1 ml cuvette. The solution was gently stirred, and absorbance at 500 nm was recorded in kinetic mode using a Cary-Varian UV–Vis spectrophotometer, as described by Pagès et al, 2008. Swelling rates were calculated from the initial slope of the absorbance trace using $\Phi = (1/A_i)*dA/dt$ and averaged across at least two independent experiments, as previously reported (2).

Dynamic Light Scattering

In the below measurements, unilamellar liposomes using 1 mg/ ml of POPC were prepared using buffer solution 5mM KCl, pH-6.3 in water. In brief, measurements using 1-15 times dilutions were conducted at 20 °C in disposable cuvettes on a Malvern HTTPS Particle Sizer in backscattering configuration with a fixed scattering angle of 173°. Dynamic light scattering revealed a homogeneous liposome preparation. The size distributions of the obtained liposomes were determined by using a Zetasizer Nano (Malvern Instruments) and found to have a major peak at ~100 nm in diameter. This finding was in line with the preparation procedure for unilamellar liposomes, even if not all liposomes may be unilamellar. To reach the equilibrium, passive diffusion allowed on average one Ptm molecule to enter and the kinetic was likely limited by entry through the porin. The detailed protocol was followed as described in (3).

Zeta Potential Measurements

The same liposome preparations used for DLS were analyzed for zeta potential, reflecting contributions from both surface charge and the Donnan potential. Aliquots (1 ml) were transferred to disposable capillary cells (Malvern Instruments), and measurements were performed on a Malvern Zetasizer Nano Z at an applied electric field strength of 10 mV/cm.

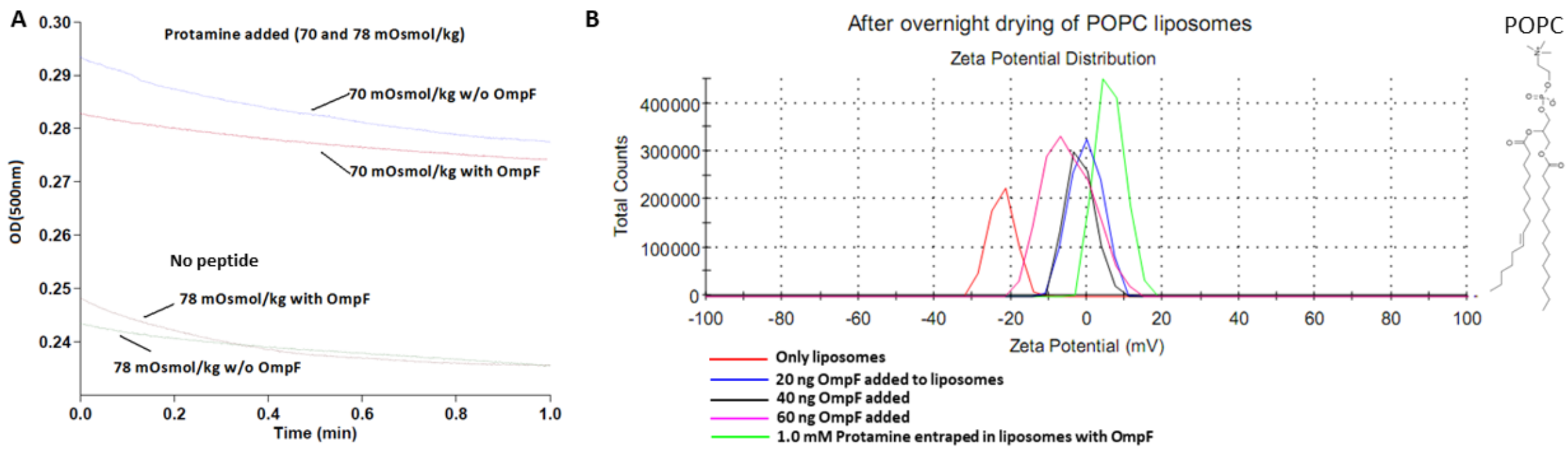

Figure S5. Size determination of the liposomes obtained by dynamic light scattering (DLS) and Zeta potential measurements. (**A**) Liposomes (2.0 mM POPC lipid) reconstituted with OmpF porin showed increase in optical density (swelling rate) after addition of Protamine. Diffusion rate of protamine across liposomes with OmpF was not high. The average size (diameter) of the liposomes was 97 nm, with a uniform size distribution that was desirable for kinetic investigations by tandem membrane assay later. (**B**) Slight increase in liposomes size integrated with OmpF size (30 times dilution factor) was observed after addition of Protamine. Small difference in Zeta potential in comparison of control liposomes with encapsulated liposomes with OmpF.

Membrane Tandem Assay

P-sulfonatocalix[4]arene (CX4), N,N' –dimethyl-9,9' –biacridinium dinitrate (LCG), Triton X-100, octyl-polyoxyethylene (O-POE), protamine sulfate, and sodium phosphate were obtained from Sigma-Aldrich. POPC (1-palmitoyl-2-oleoyl-sn-glycero-3-phosphocholine; 25 mg/mL in chloroform) was purchased from Avanti Polar Lipids, and NAP-25 desalting columns from GE Healthcare. Purified OmpF (2 mg/ml in 1% O-POE) was used for proteoliposome preparation.

The CX4/LCG reporter assay was performed as previously optimized (4). Briefly, a lipid film was generated by depositing 100 µl of POPC stock solution (25 mg/ml) in a 5 ml round-bottom flask, followed by slow nitrogen drying and overnight vacuum desiccation. The film was hydrated with 1 ml of buffer containing 10 mM NaH_2PO_4, 700 µM CX4, and 500 µM LCG, and gently stirred on a rotary evaporator for ~20 min at room temperature. The resulting suspension underwent multiple times freeze-thaw cycles to generate unilamellar liposomes. Encapsulated CX4/LCG liposomes were separated from free dye using NAP-25 size-exclusion chromatography under identical buffer conditions (4). Removal of non- entraped host and dye was done by dialysis.

For protamine and short-peptide uptake assays, 20 µl of CX4/LCG-loaded liposomes (25 µM lipid) in 10 mM sodium phosphate buffer (pH 7.0, 25 °C) were placed in a 2 ml cuvette, and time-resolved fluorescence was recorded. OmpF was added to a final concentration of 45 nM (2.8 µl of 2 mg/ml stock) to generate proteoliposomes in situ. At the end of each kinetic trace, Triton X-100 (50 µl of 1% solution) was added to lyse vesicles and determine maximal fluorescence. Fluorescence measurements were performed on a Varian Eclipse spectrofluorimeter using excitation at 367 nm and emission at 500 nm. Experimental details followed (3).

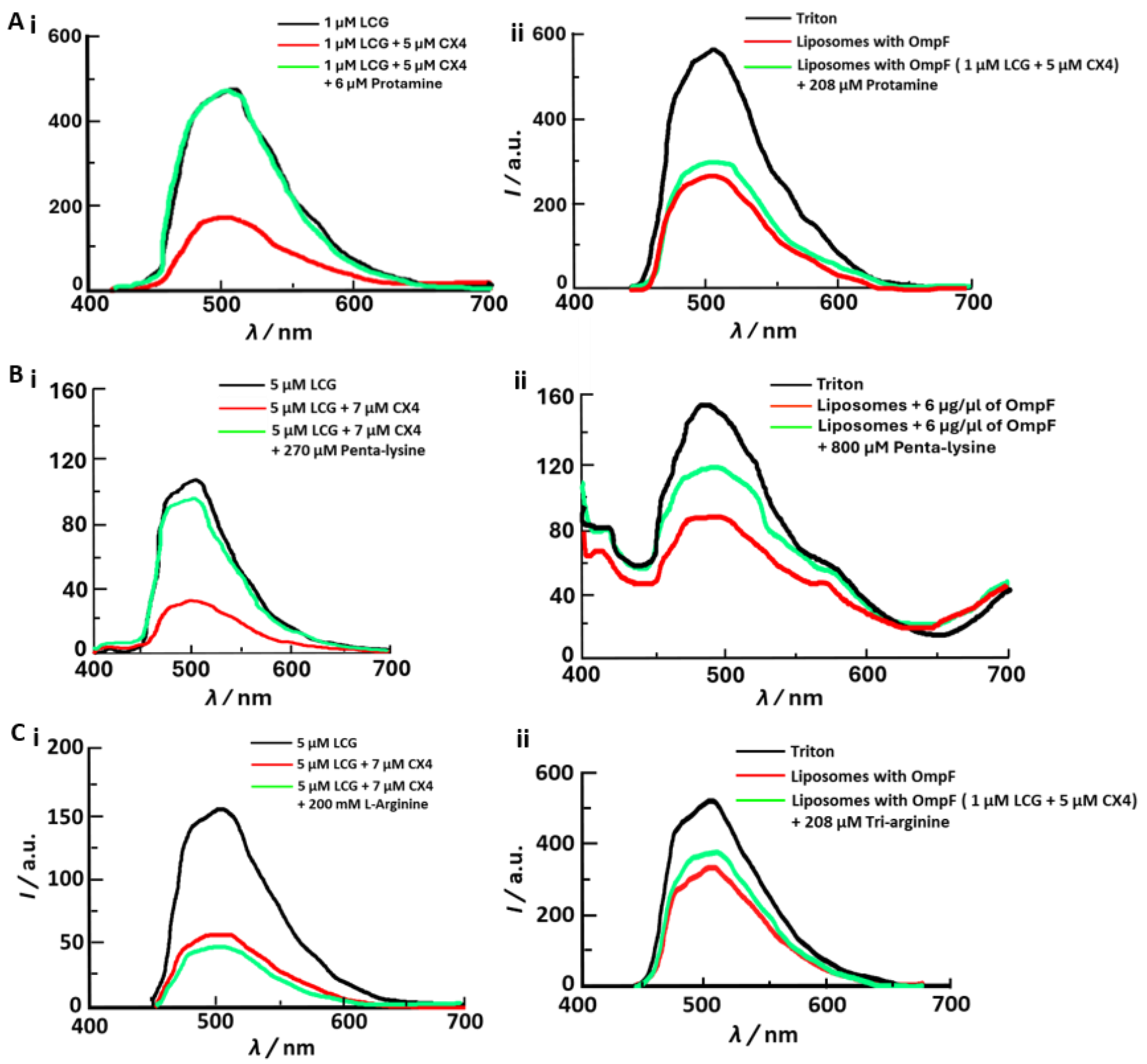

Figure S6. Substrate selective supramolecular tandem assay using ideal host-dye reporter pair and cationic peptides. (**A-C, i**) Fluorescence signal showed efficient displacement of guest LCG strongly fluorescent fluorophore dye (black trace) from macrocyclic host CX4 cavity (red trace) by competitors such as Protamine, and higher concentration of Penta-lysine and Arginine (green trace) in solutions containing guest-host complex without liposomes integrated with OmpF channels. (**A-C, ii**) Solutions with liposomes reconstituted with OmpF (red trace) and encapsulated host-dye treated with detergent 2% Triton-X as a control (black trace) had no effect on liposomes. The host CX4 was able to diffuse through the SUVs liposomes membrane. The fluorescence intensity of the guest dye LCG changed upon encapsulation by the host showing entrapment of host-guest. Higher concentration of competitors peptides such as Protamine, Penta-lysine and Tri-arginine appeared to permeate into the liposomes through the OmpF channels by displacing the LCG dye from its host CX4 complex which produced an increase in fluorescence intensity (green trace) in 10 mM sodium phosphate solution, pH 7.0.

References

1. Nikaido H, Rosenberg EY. Porin Channels in Escherichia coli: Studies with Liposomes Reconstituted from Purified Proteins. J Bacteriol. 1983;153:241-52.
2. Pages JM, James CE, Winterhalter M. The porin and the permeating antibiotic: a selective diffusion barrier in Gram-negative bacteria. Nat Rev Microbiol. 2008;6:893-903.
3. Pangeni S, Prajapati JD, Bafna J, Nilam M, Nau WM, Kleinekathöfer U, et al. Large-Peptide Permeation Through a MembraneChannel: Understanding Protamine Translocation Through CymA from Klebsiella Oxytoca. Membrane Channels. 2021;60:8089-94.
4. Ghale G, Lanctot AG, Kreissl HT, Jacob MH, Weingart H, Winterhalter M, et al. Chemosensing ensembles for monitoring biomembrane transport in real time. Angew Chem Int Ed Engl. 2014;53:2762-5.
